# Editorial: Genetics of thyroid gland

**DOI:** 10.3389/fgene.2023.1219472

**Published:** 2023-05-30

**Authors:** Tatijana Zemunik, Mirjana Babić Leko, Ivana Gunjača

**Affiliations:** Department of Medical Biology, School of Medicine, University of Split, Split, Croatia

**Keywords:** thyroid gland, genetics, thyroid-associated ophthalmopathy, resistance to thyroid hormone, thyroid diseases

Normal human physiology is vitally influenced by thyroid hormones. Thyroid hormones affect almost all human tissues influencing development and growth, regulating vital body functions and metabolism (Panicker, 2011). The prevalence of thyroid diseases is up to 10% in the general population and represents a significant health problem (Panicker et al., 2010). Thyroid hormones/antibodies are considered complex traits and their concentrations are influenced by genetic and environmental factors. However, genes responsible for thyroid hormone/antibody concentrations as well as environmental factors remain largely undetermined (Panicker, 2011). In the last few decades, advanced high-throughput sequencing technologies have contributed to the development of genome-wide association studies (GWAS) which rapidly become the most used method for the identification of common genetic variants associated with complex traits and diseases (Chimusa and Defo, 2022). Furthermore, GWAS meta-analysis represents a statistical synthesis of multiple independent GWAS studies and consequently increases power and excludes false positive variants. Therefore, a GWAS meta-analysis has grown into a significant statistical method for the identification of new genetic loci underlying complex traits and diseases (Evangelou and Ioannidis, 2013). A fundamental goal of genetic studies is to predict complex phenotypes from genomic data, and prediction studies become very popular recently (Morgante et al., 2018). The best-investigated complex phenotypes of the thyroid gland, as well as thyroid diseases at the whole genome level, are thyroid-stimulating hormone (TSH), free thyroxine (fT4) levels, Graves’ disease and thyrotoxicosis (Zhou et al., 2020; Teumer et al., 2018; Sakaue et al., 2021; Backman et al., 2021).

Although the mentioned approaches are very important in determining the genetic function of the thyroid gland, there are other types of genetic studies that help in elucidating the function of the gland. One of the manuscripts published in this Research Topic aimed to identify ferroptosis-related genes (FRGs) that may have a diagnostic and therapeutic association with thyroid-associated ophthalmopathy (TAO). Using a high-throughput gene expression database (GEO) authors identify differentially expressed genes and differentially expressed FRGs between TAO patients and controls. The authors also applied immune cell infiltrative analysis using the CIBERSORT algorithm to prove the difference between TAO patients and controls, and finally, they also identify differentially expressed ferroptosis-related lncRNAs in the TAO group. Gained results were validated by *in vitro* experiment analysing FRGs and lncRNAs in orbital fibroblasts of three TAO patients and three healthy individuals (Chen et al.). Another manuscript found digenic variants (of five analysed pathogenic genes) in five individuals affected with congenital hypothyroidism (CH). In addition, seven novel genetic variants were identified in these patients (Yang et al.). Resistance to thyroid hormone (THR) is a rare hereditary disorder caused by mutations in the thyroid hormone receptor beta (THRβ) gene. Two case reports published in this Research Topic deal with the problem of this syndrome. In the first one, authors sequenced the coding region of THRβ gene and found a novel dinucleotide substitution located in codon 453 in the affected woman with coexisting autoimmune thyroid disease (Skowrońska-Jóźwiak et al.). In the second one, the authors reported two cases of THRβ gene mutation with coexisting papillary thyroid carcinoma (PTC). After reviewing the literature, the authors state that 17 cases of THRβ gene mutation coexisting with PTC have been described to date (Fang et al.).

All manuscripts published in this Research Topic contribute to the elucidation of the genetic background of thyroid gland dysfunction.
